# Some Valuable Notes for Practitioners

**Published:** 1921-04-09

**Authors:** 


### SURGERY IN OTITIS CASES.

### Some Valuable Notes for Practitioners.

The ear is such a complicated series of bony chambers and corridors, furnished in so complex a manner, that its surgery chiefly belongs to the domain of the consulting aurist. Nevertheless, the general practitioner is able to carry out a certain amount of surgical treatment without the aid of expert advice, and as he may, at any time, be placed in such a position that he is entirely dependent upon his own resources, it is of the utmost importance that he should be on somewhat intimate terms with this branch of surgery. Of the lesions associated with the external ear perhaps the most important are the following (in this classification we will chiefly confine ourselves to surgical treatment).

A. Excessive accumulation of cerumen. This causes a variety of symptoms, such as deafness, tinnitus, "rushing" in character, and sometimes pain and vertigo. To remove this first saturate the wax with hydrogen peroxide 10 vols., or even 20 if very hard and of long-standing; then gently syringe with a bland alkaline solution (such as bicarbonate of soda), using a metal syringe for preference.

B. Acute perichondritis of pinna. This is generally secondary to some septic lesion in the external auditory meatus. Remember that the cartilage of the meatus is directly continuous with that of the pinna. If suppuration has occurred or is about to occur, make free incisions, following the varying curves of the auricle so far as is possible; the chief difficulty will lie in the prevention of the after-formation of excessive scar tissue, resulting in a distorted and unsightly pinna.

C. Acute furunculosis of the external auditory meatus. Here we have a condition producing excruciating pain and often associated with pre-auricular, and perhaps post-auricular, meatal tenderness, swelling, and œdema. Make a free incision into the most pointing area, or areas, using a general anæsthetic; this will be most successful in relieving pain at once, and should provide an adequate drainage; then syringe the meatus with Eusol and plug lightly with iodoform gauze wrung out of Eusol.

D. Chronically recurring furunculosis of the external auditory meatus is not uncommon, and is often most difficult to cure. Autogenous vaccine, combined with free drainage, and iodoform, Eusol, and antiphlogistine, used as stated above, form a very good treatment, but every means should be taken to raise the general resisting power of the patient as well.

E. acute otitis media suppurativa, without perforation of the tympanic membrane. Here again the pain is very acute, and the sufferer will insistently demand early relief. This can be done by making a curved incision, with a very sharp and specially shaped scalpel, into the most prominent part of the membranic swelling, which is generally situated in the posterior quadrant. It will be necessary to administer a general anæsthetic, for no local application is of any real service. There is no doubt that early myringotomy is by far the best treatment, for not only is the pain instantly relieved, but the edges of the perforation are not ragged as is the case when the abscess bursts spontaneously, and therefore heals up rapidly, and very often with no loss of hearing.

F. Sometimes acute otitis media is associated with acute furunculosis, and in these cases great care must be taken to ensure that there is free drainage from the middle ear. It is often very difficult to see the membrana tympani at all, owing to the swelling of the external meatus, but if there should be clinical evidence of involvement of the middle ear, then the membrane should be inspected under nitrous oxide, and incised if necessary.

It is well to remember that acute external otitis may cause œdema, pain, and tenderness all round the pinna which may closely simulate that due to acute mastoiditis, but in the former condition the tenderness is more in relation to the pinna itself than to the mastoid bone, and is therefore more superficial in position; again, the two lesions may co-exist, and here the diagnosis will be very greatly helped if a purulent discharge can be seen emerging in a pulsating manner from the depths of a swollen external meatus, for in these circumstances the middle ear is almost certainly involved.

### Lesions of Middle Ear.

G. Acute mastoid suppuration; here, in a large majority of cases, surgical interference is demanded, and seeing that the mastoid antrum is involved, the surgeon should always provide a free drainage for that cavity, and then explore and lay open any other areas which are in need of such measures.

H. Chronic mastoid suppuration; this generally involves a radical mastoid operation which, being a serious and often most difficult proceeding, necessitates the help of a specialist.

I. Removal of polypi in the external auditory meatus; polypi are usually the result of chronic suppuration, and in this situation, in the vast majority of cases, proceed from the middle ear, it is therefore important to remember that though the removal may be beneficial by providing a freer drainage for a suppurating cavity, yet, as the cause is still present, they will recur. Also that their removal is not unattended with risk, for not only has a facial paralysis immediately followed the operation, but also serious mastoid symptoms have supervened.

Summarising the above remarks, it cannot be urged too strongly that no surgeon should undertake such delicate and serious operations unless provided with good experience and special skill, besides possessing the necessary instruments.

There are, however, many occasions when a general practitioner in a country district may be faced with a problem which he alone can solve. To allow a tympanic membrane to rupture spontaneously is bad surgery, but a faulty myringotomy may also lead to serious consequences.

Perhaps no aural operation is productive of better results than successful removal of cerumen, which is within the scope of any practitioner; incision and drainage of external otitic suppuration need not necessarily call for the help of a specialist, unless the circumstances suggest such a procedure. Finally, the drainage of the mastoid antrum and cells in a straightforward case of acute suppuration presents no special difficulties provided that certain anatomical details are remembered.

